# Integrating Chlorophyll Fluorescence with Anatomical and Physiological Analyses Reveals Interspecific Variation in Heat Tolerance Among Eight *Rhododendron* Taxa

**DOI:** 10.3390/plants14233664

**Published:** 2025-12-01

**Authors:** Wenfang Guo, Jiaxin Wei, Hao Yu, Yurui Wang, Jingli Zhang, Shusheng Wang

**Affiliations:** 1 Jiangxi Provincial Key Laboratory of Plant Germplasm Innovation and Genetic Improvement, Lushan Botanical Garden, Chinese Academy of Sciences, Jiujiang 332900, China; guowenfang1986@163.com (W.G.); 17719244228@163.com (J.W.); 18668707768@163.com (H.Y.); wyrplant@163.com (Y.W.); 2 Department of Pomology, College of Agronomy, Yunnan Agricultural University, Kunming 650030, China

**Keywords:** *Rhododendron*, high-temperature stress, photosystem II, physiological characteristics, comprehensive evaluation

## Abstract

To investigate interspecific variation in heat tolerance and underlying adaptation mechanisms in *Rhododendron*, three-year-old potted seedlings of eight taxa, representing four subgenera within the genus *Rhododendron*, were subjected to 40 °C high-temperature stress. Heat tolerance was comprehensively assessed using phenotypic observation, chlorophyll fluorescence imaging, microscopic examination, and physiological measurements. Results revealed that leaf damage in *Rhododendron oldhamii* and *Rhododendron × pulchrum* reached grade III, whereas *Rhododendron latoucheae* exhibited only grade II injury with rapid recovery. Chlorophyll fluorescence analysis showed a significant decrease in the maximum quantum efficiency of PSII (F_v_/F_m_) in *R. liliiflorum* and *R. × pulchrum*, followed by rapid recovery, while *R. latoucheae* maintained stable F_v_/F_m_ values. Stomatal closure occurred in all taxa post-stress; stomatal characteristics of *R. liliiflorum* and *R. simiarum* remained stable, and leaf tissue structure was least affected in *R. kiangsiense*. *R. × pulchrum* demonstrated the most pronounced structural recovery. Physiologically, *R. oldhamii* exhibited the greatest increases in electrolyte leakage (EL) and malondialdehyde (MDA) content. *R. simiarum* accumulated the highest proline content under stress, while *R. latoucheae* showed the most significant proline reduction during recovery. By integrating multiple indicators through principal component analysis (PCA) and a membership function, and assigning weights based on variance contribution, the heat tolerance was comprehensively evaluated and ranked as follows: *R. latoucheae* > *R. simiarum* > *R. oldhamii* > *R. ovatum* > *R. fortunei* > *R. liliiflorum* > *R. kiangsiense* > *R. × pulchrum*. These findings demonstrate significant differences in heat tolerance among *Rhododendron* taxa at the subgenus level, with the subgenus *Azaleastrum* generally possessing stronger short-term heat tolerance compared to the subgenus *Tsutsusi*. This study provides a theoretical basis for heat-tolerant cultivar breeding and landscape application of *Rhododendron*.

## 1. Introduction

*Rhododendrons* (*Rhododendron* spp.) are widely used in landscaping due to their excellent ornamental value. However, their preference for cool, moist environments and intolerance to high temperatures have long limited their growth, development, geographical distribution, and promotional value [1,2]. Therefore, studying the mechanisms of high-temperature response and breeding new *Rhododendron* varieties with stronger tolerance is crucial.

Chlorophyll fluorescence imaging is a non-destructive, rapid, and highly sensitive technique that reflects the real-time functional status of plant photosynthesis and enables early diagnosis of environmental stress before visible symptoms occur [3]. Previous studies have primarily applied this technique to assess cold and heat stress in plants [4,5,6]. In recent years, its applications have expanded to include light stress [7], drought [8,9], salinity stress, and heavy metal contamination [10]. High-temperature stress primarily impairs photosynthetic electron transport and reduces PSII activity, thereby weakening overall photosynthetic performance [11]. Consequently, chlorophyll fluorescence parameters are widely used to assess heat tolerance in plants [12,13]. For example, studies in tomato demonstrated that 40 °C for 24 h exerts a more pronounced impact on the PSII electron transport chain than low-temperature stress, and that reproductive tissues are more sensitive than leaves, highlighting the ability of chlorophyll fluorescence to discriminate the site and severity of temperature-induced damage [14]. Research on wheat further showed that detached leaves exhibited a rapid decline in the maximum quantum efficiency of PSII (F_v_/F_m_) after 2 h at 40 °C or 30 min at 45 °C, with responses faster and stronger than those observed in intact plants exposed to 40 °C for three days, indicating tissue-specific differences in PSII sensitivity under various heat regimes [15]. In maize, F_v_/F_m_ remained near normal under hot-wind stress, but both F_m_ and F_v_ declined significantly, accompanied by increased NPQ and reduced ΦPSII, suggesting that NPQ and ΦPSII serve as sensitive indicators for distinguishing genotypic variation in heat tolerance [16].To date, heat-stress research on *Rhododendron* taxa has largely focused on molecular-level responses [17,18], whereas studies employing chlorophyll fluorescence imaging remain relatively limited.

Plant tolerance to abiotic stress is often associated with multiple physiological indicators. For instance, high-temperature stress can inhibit photosynthesis in tomato leaves, affect carbohydrate accumulation and transport, and disrupt the reactive oxygen species (ROS) scavenging system [19,20]. Corn and blueberries respond to high-temperature stress by regulating stomatal aperture and leaf microstructure to reduce leaf temperature [21,22]. Heat-tolerant wheat varieties accumulate more proline than heat-sensitive varieties to reduce osmotic potential and protect cells [23]. Additionally, other studies have shown significant differences in heat tolerance between heat-tolerant wild grape varieties and heat-sensitive European grape varieties [24]. By crossing heat-sensitive wild hairy kiwifruit with heat-tolerant Chinese kiwifruit, a genetic population was thus established to produce new heat-tolerant varieties [25]. Therefore, the physiological response mechanisms of different taxa to high-temperature stress are different, whereas heat tolerance is significantly related to genetic characteristics. However, comparative physiological studies across *Rhododendron* subgenera under standardized high-temperature stress conditions have not been reported.

This research compared the heat tolerance differences among eight *Rhododendron* taxa distributed at different altitudes from four *Rhododendron* subgenera through multidimensional analysis of chlorophyll fluorescence imaging, stomatal morphology, leaf tissue anatomy, and physiological characteristics. It further revealed the adaptive mechanisms of different *Rhododendrons* in response to high-temperature stress. This analysis provides future reference for *Rhododendron* heat tolerance breeding and horticultural application.

## 2. Results

### 2.1. Effects of Heat Stress on Leaf Phenotype in Eight Rhododendron Taxa

**Figure 1:**
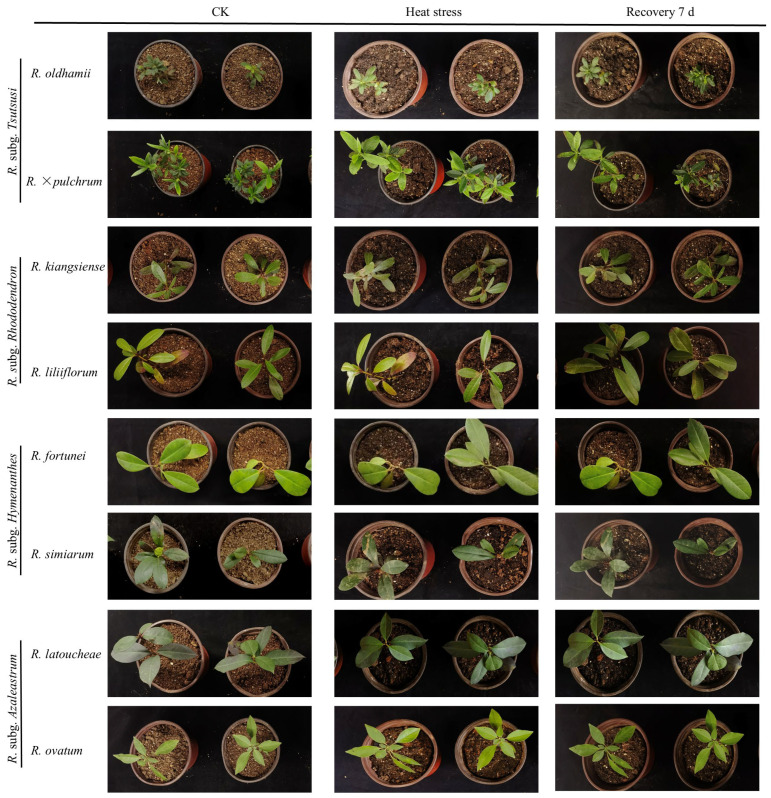
The phenotypes of eight *Rhododendron* taxa under control, heat stress, and after 7 d recovery.

Figure 1 shows the plant phenotypes under high-temperature stress. *R. oldhamii* and *R. × pulchrum* were the most sensitive plants, showing wilting, drooping, and shriveling of 40% of their leaves when exposed to 40 °C high-temperature stress, with damage severity reaching Grade III. They also had the weakest recovery capacity (damage severity remained at Grade III even after 7 d of recovery). *R. kiangsiense* and *R. liliiflorum* exhibited wilting and drooping in 20% of their leaves after high-temperature stress, with damage severity reaching Grade II. After 7 d of recovery at a normal temperature, the damage severity decreased to Grade I, indicating a better recovery capacity (Table 1). *R. fortunei* and *R. simiarum* exhibited Grade II leaf damage under high-temperature stress. When transferred to normal temperatures for recovery cultivation, some wilted leaves recovered by the 4th day, with the damage reducing to Grade I. This indicates a strong recovery capacity. *R. latoucheae* and *R. ovatum* also exhibited Grade II damage under high-temperature stress. However, after being transferred to normal temperature conditions, the leaf damage rapidly recovered to Grade I (Table 1). Thus, it is evident that different *Rhododendron* exhibit distinct leaf phenotypes in response to high-temperature stress.

### 2.2. Effects of Heat Stress on Photosynthetic Performance in Eight Rhododendron Taxa

**Figure 2:**
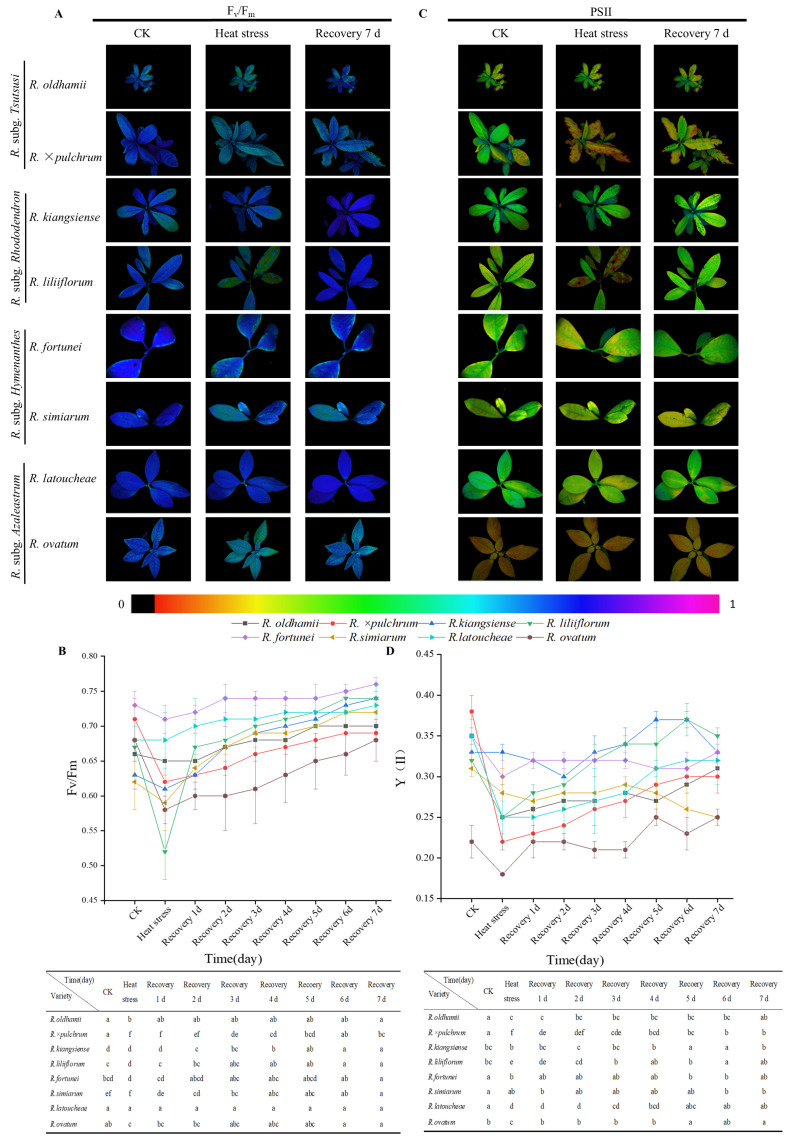
Chlorophyll fluorescence responses of eight *Rhododendron* taxa under control, heat stress, and recovery conditions. (**A**,**C**) Chlorophyll fluorescence images F_v_/F_m_ and Y(II) of eight *Rhododendron* taxa under control conditions, after 24 h of heat stress, and following 7 d of recovery. (**B**,**D**) Temporal changes in F_v_/F_m_ and Y(II) of the eight taxa during heat stress and the 7 d recovery period. Data represent mean ± SD (*n* = 3). Different letters indicate significant differences among taxa at each time point based on one-way ANOVA followed by Duncan’s multiple range test (*p* < 0.05).

As shown in Figure 2A,B, the F_v_/F_m_ indices of *R. liliiflorum*, *R. × pulchrum*, and *R. ovatum* were significantly lower than before treatment, while the other five taxa showed no significant change. After 7 d of recovery, the F_v_/F_m_ values of the three aforementioned *Rhododendron* taxa gradually returned to pre-treatment levels. *R. liliiflorum* recovered the fastest, achieving full recovery in 3 d. Figure 2C,D show that the PSII index decreased rapidly and significantly in *R. liliiflorum*, *R. × pulchrum*, and *R. fortunei* after high-temperature stress. After 7 d of recovery at normal temperature, *R. × pulchrum* and *R. liliiflorum* recovered to pre-stress levels. Notably, the PSII indices of *R. simiarum* and *R. latoucheae* remained significantly higher than the control group, showing a continuous downward trend even after 7 d of recovery. These results suggest that high temperatures generally impair the photosynthetic function of *Rhododendrons*, with significant interspecific variations observed in heat sensitivity and recovery capacity.

As illustrated in Figures S1–S3, heat stress resulted in a significant decrease in the photochemical quenching (qP) coefficient across seven *Rhododendron* taxa compared to the control. Meanwhile, the electron transport rate (ETR) exhibited a general downward trend. The most substantial decline in qP was observed in *R. latoucheae* and *R. ovatum*, while the greatest reduction in ETR was observed in *R. × pulchrum* (Figures S1A,B and S3C). Concurrently, the non-photochemical quenching coefficient (qN) and the quantum yield of regulated energy dissipation Y(NPQ) increased significantly in most taxa. Additionally, the quantum yield of non-regulated energy dissipation, Y(NO), and non-photochemical quenching (NPQ) increased markedly in all taxa, peaking after 24 h of stress (Figures S1C,D, S2A–D and S3A,B). After a 7 d recovery period under normal temperature conditions, the qP values for *R. liliiflorum*, *R. simiarum*, and *R. × pulchrum*, as well as the ETR values for *R. liliiflorum* and *R. ovatum*, gradually returned to control levels. In contrast, the qP values of *R. latoucheae*, *R. ovatum*, and *R. fortunei* remained significantly suppressed. Although NPQ, qN, and Y(NPQ) trended downward during recovery across the eight taxa, they remained significantly elevated above control levels after 7 d. However, the Y(NO) index decreased rapidly upon returning to normal temperature, with some taxa recovering to baseline levels within 7 d. Conversely, *R. oldhamii* showed no significant changes in its qN and Y(NPQ) values. In conclusion, these photosynthetic parameters accurately and promptly indicate the extent of heat stress in rhododendrons and their subsequent recovery capacity.

### 2.3. Impact of Heat Stress on Stomatal Traits in Eight Rhododendron Taxa

**Figure 3:**
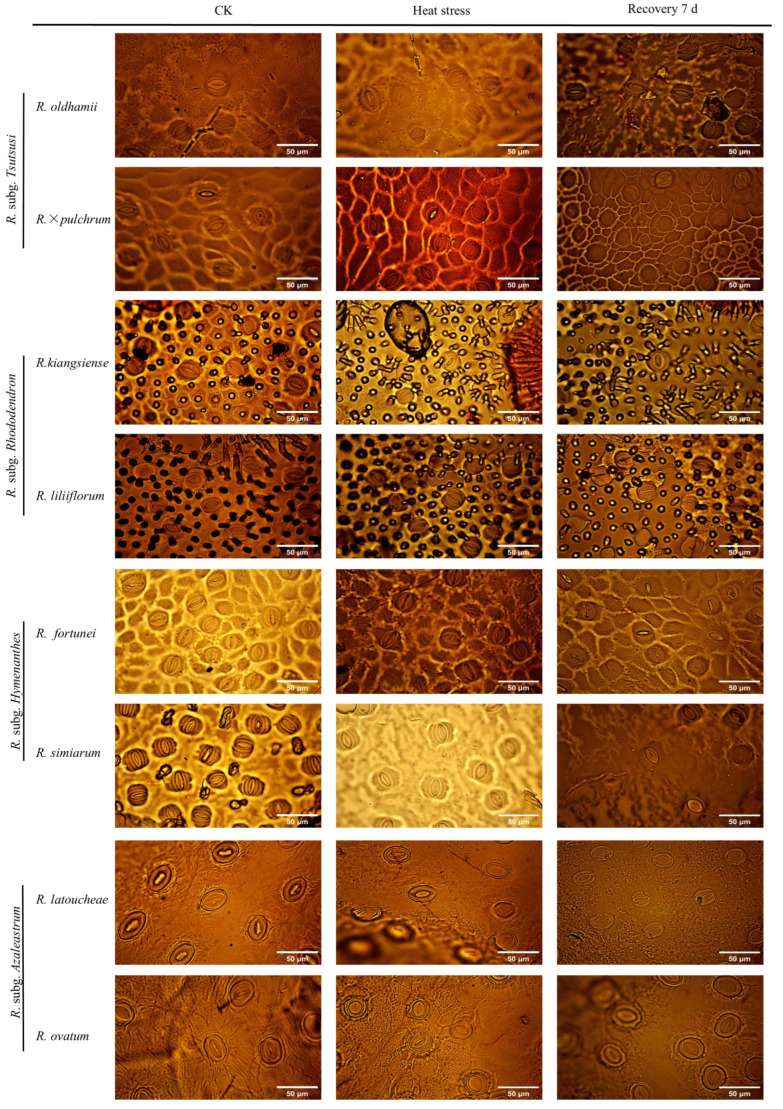
Ultrastructure of leaf epidermal cells of eight *Rhododendron* taxa under control, heat stress, and after 7 d of recovery. The analysis focuses on stomatal morphology, comparing heat stress-induced changes in stomatal characteristics among taxa. Scale bar = 50 μm.

Stomatal characteristics of leaves under high-temperature stress are shown in Table 2 and Figure 3. Stomatal length decreased post-stress in all cultivars, with the most substantial reductions occurring in *R. oldhamii*, *R. × pulchrum*, and *R. latoucheae.* Upon transfer to ambient temperature for recovery, stomatal length in *R. oldhamii*, *R. × pulchrum*, and *R.latoucheae* exhibited a significant rebound, whereas it remained stable in *R. liliiflorum* and *R. simiarum* throughout the experimental period. All eight *Rhododendron* taxa displayed pronounced stomatal closure under heat stress. The most significant reduction in stomatal width was observed in *R. fortunei*, followed by *R. liliiflorum* and *R. oldhamii*. During the recovery phase under ambient conditions, stomata reopened. Notably, *R. simiarum* showed a more marked recovery in stomatal width, and *R. liliiflorum* fully recovered to control levels within 7 d. In terms of stomatal area, a significant decrease was observed across all cultivars, with *R. kiangsiense* and *R. latoucheae* experiencing the greatest declines. Conversely, stomatal density increased following stress in all taxa, with the highest increases recorded in *R. × pulchrum* and *R. liliiflorum*. After 7 d of recovery, stomatal density declined in most cultivars. While *R. liliiflorum* and *R. ovatum* returned to control levels, *R. × pulchrum* maintained an elevated density that was 16.7% higher than the control (Table 2). Collectively, these findings indicate that *R. liliiflorum* and *R. simiarum* exhibited greater stability in their stomatal characteristics, as evidenced by minimal changes in response to heat stress.

### 2.4. Divergent Responses of Leaf Anatomical Structures to Heat Stress in Eight Rhododendron Taxa

**Figure 4:**
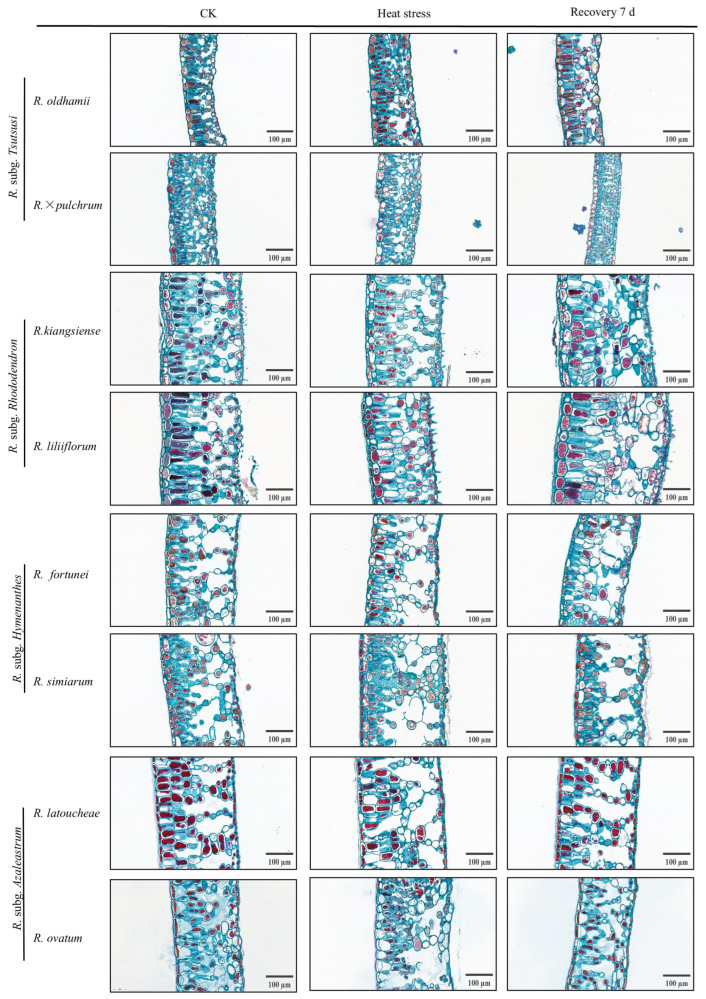
Leaf tissue structure images of eight *Rhododendron* taxa under control (CK), heat stress, and after 7 d of recovery. Scale bar = 100 μm. Sections show variations in mesophyll and epidermal structure across taxa.

As illustrated in Table 3 and Figure 4, the leaf tissue structures of different *Rhododendron* taxa exhibited distinct alterations under high-temperature stress. All eight taxa showed significant reductions in upper and lower epidermal thickness. Notably, *R. ovatum*, *R. fortunei*, and *R. oldhamii* exhibited more pronounced decreases in the upper epidermis, while *R. liliiflorum*, *R. simiarum*, and *R. × pulchrum* experienced more severe shrinkage in the lower epidermis. All taxa exhibited a significant increase in palisade tissue thickness due to high-temperature stress in comparison to the control group, while spongy tissue thickness showed a substantial decrease. Consequently, the palisade to spongy tissue thickness ratio was significantly higher under stress than in the control group. *R. × pulchrum* exhibited a larger increase in this ratio among the studied taxa, while R. kiangsiense showed the least change. After 7 d of recovery at normal temperatures, the thickness of both epidermal layers generally increased, with *R. liliiflorum* returning to control levels. Palisade tissue thickness significantly decreased, while spongy tissue thickness evidently recovered. *R. × pulchrum* exhibited the most pronounced changes before and after stress, as well as the greatest variation in the ratio of palisade to spongy tissue during recovery. This indicates its heightened sensitivity to structural modifications induced by high temperatures and its strong regenerative capacity. *R. liliiflorum* was the second most responsive taxon. In contrast, *R. kiangsiense* exhibited relatively stable tissue thickness variations and showed no significant difference from the control (Table 3).

### 2.5. Impact of Heat Stress on Rhododendron Physiology

**Figure 5:**
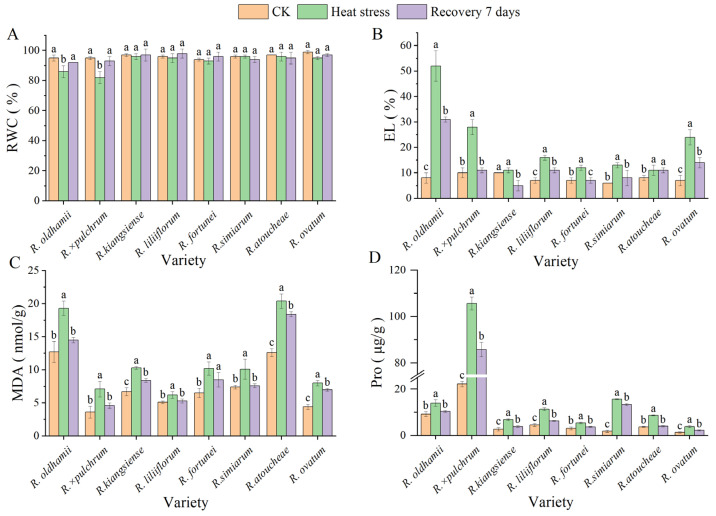
Physiological and biochemical indicators of eight *Rhododendron* taxa under control (CK), heat stress, and recovery. (**A**) Relative water content (RWC); (**B**) electrolyte leakage (EL); (**C**) malondialdehyde (MDA) content; (**D**) proline (Pro) content. Data are mean ± SD (*n* = 3). Different lowercase letters indicate significant differences among taxa under the same treatment (*p* < 0.05).

As shown in Figure 5, heat stress induced a decline in RWC in *R. × pulchrum* and *R*. *oldhamii* by 13% and 10%, respectively. After a 7 d recovery period under ambient conditions, the RWC of these two taxa returned to control levels. The other taxa did not exhibit significant changes (Figure 5A). All eight taxa showed a significant increase in EL after being subjected to stress. Transferring them to a normal temperature for 7 d facilitated a marked recovery in all *Rhododendrons*. The most pronounced change occurred in *R. oldhamii*, followed by *R. × pulchrum*; the remaining five strains showed more modest alterations (Figure 5B). MDA content, which is an indicator of lipid peroxidation, accumulated substantially in all strains following stress, with the most significant elevations occurring in *R. oldhamii* and *R. ovatum*. After waiting 7 d, there was a lot less MDA in the plants. *R. liliiflorum*, *R. simiarum*, *R. × pulchrum*, and *R. oldhamii* went down to normal levels (Figure 5C). Pro content also exhibited a significant accumulation in response to stress, particularly in *R. simiarum* and *R. × pulchrum*. Upon recovery, proline levels decreased significantly across all taxa. The most substantial reduction was in *R. latoucheae*, which, together with *R. fortunei*, recovered to control levels (Figure 5D). We observed that high-temperature stress differentially impacted the physiological characteristics of various rhododendron germplasms. A 7 d recovery period was sufficient for substantial repair in most taxa, among which *R. ovatum* showed the strongest physiological recovery ability.

### 2.6. Comprehensive Evaluation of Heat Tolerance in Rhododendron

**Figure 6:**
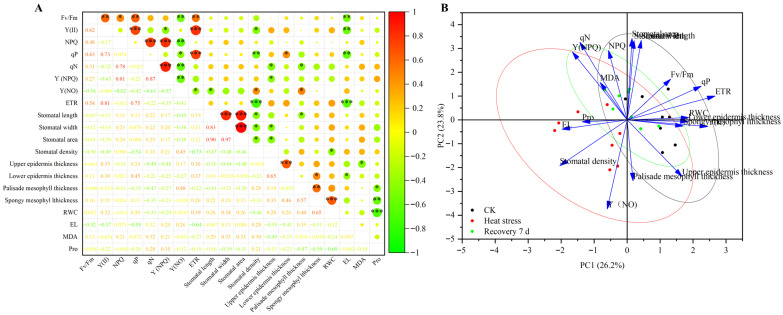
(**A**) Correlation matrix showing Pearson correlation coefficients among the measured physiological parameters in eight *Rhododendron* taxa under control, heat stress, and 7 d recovery conditions. Circle size and color scale indicate the strength and direction of correlations. Note: Significance levels are denoted as follows: * *p* < 0.05, ** *p* < 0.01, and *** *p* < 0.001. (**B**) Principal component analysis (PCA) score-loading biplot illustrating the distribution of samples in response to control, heat stress, and recovery treatments. Ellipses indicate the 95% confidence intervals for each treatment group.

Pearson correlation analysis was performed on the photosynthetic function, stomatal characteristics, leaf anatomical structure, and physiological indicators of eight *Rhododendron* taxa before and after heat stress. The results are shown in Figure 6A; F_v_/F_m_ was positively correlated with PSII, NPQ, qP, and ETR in the photosynthetic function indicators, negatively correlated with Y(NO) and EL, and showed no significant correlation with other indicators. PSII was extremely significantly positively correlated with qP and ETR among the photosynthetic function indicators and negatively correlated with stomatal density and EL. NPQ was positively correlated with qN, NPQ with Y(NPQ), and qP with ETR and Y(NO). NPQ was negatively correlated with Y(NO) and qP with stomatal density and EL. ETR was extremely significantly negatively correlated with stomatal density and EL. Stomatal length was highly significantly positively correlated with stomatal width and area, but negatively correlated with stomatal density and palisade tissue thickness. Stomatal width was highly significantly positively correlated with area, but negatively correlated with stomatal density and epidermal layer thickness, without a significant relation to other indicators. Stomatal density was negatively correlated with leaf RWC. Leaf epidermal layer thickness was highly significantly positively correlated with hypodermal layer thickness and negatively correlated with the physiological indicator MDA accumulation. Palisade tissue thickness was positively correlated with spongy tissue thickness but negatively correlated with Pro accumulation. Leaf spongy tissue thickness was positively correlated with RWC but negatively correlated with Pro accumulation. RWC was highly significantly negatively correlated with Pro, while other physiological indicators showed no significant correlations (Figure 6A). The correlations between variables shown in Figure 6B are consistent with the results in Figure 6A. However, there was an obvious separation between the groups of variables measured after heat stress and those measured before stress and after recovery, while the groups of variables measured after recovery and before stress overlapped significantly (Figure 6B).

The heat tolerance indices of eight *Rhododendron* taxa were analyzed using principal component analysis (Table 4). The contribution rates of the first six principal components (eigenvalues ≥ 1) were 26.2%, 23.79%, 16.10%, 10.19%, 7.34%, and 5.33%, respectively, with a cumulative variance contribution rate of 88.98%. This indicates that these six principal components effectively represent most of the information in the original indicators. The plant’s photosynthetic electron transport efficiency and photochemical activity are primarily reflected in PC1, which has a high loading on photosynthetic parameters such as leaf ETR, PSII, and qP. PC2 has high loadings on leaf stomatal area, stomatal length, qN, and NPQ, indicating that this component primarily characterizes stomatal morphological features and non-photochemical quenching protection mechanisms. PC3 has significant loadings on stomatal aperture and leaf RWC, primarily reflecting the plant’s osmotic regulation capacity and structural adaptability. PC4 has high loadings on palisade and spongy tissue thickness. PC5 and PC6 have high loadings on the physiological indicator MDA, which is related to oxidative damage and leaf structure. These components reflect the plant’s stress tolerance and structural characteristics.

Finally, the weights of the principal components (PC1 to PC6) were determined based on the contribution rates and cumulative contribution rates of each comprehensive indicator (Table 5), with values of 0.24, 0.21, 0.19, 0.16, 0.11, and 0.09, respectively. The D-values for the eight *Rhododendron* taxa were calculated as follows: *R. latoucheae* (0.68), *R.simiarum* (0.598), *R. oldhamii* (0.555), *R. ovatum* (0.510), *R. fortunei* (0.504), *R. liliiflorum* (0.498), *R. kiangsiense* (0.440), and *R. × pulchrum* (0.401). Based on the D-values, the heat tolerance ranking of the eight *Rhododendron* taxa was *R. latoucheae* > *R. simiarum* > *R. oldhamii* > *R. ovatum* > *R. fortunei* > *R. liliiflorum* > *R. kiangsiense* > *R. × pulchrum.*

## 3. Discussion

Damage to the photosynthetic system under heat stress is a central manifestation of interspecific variation in thermotolerance. Chlorophyll fluorescence parameters, which serve as sensitive proxies for photosynthetic performance, showed significant differences among taxa. *R. liliiflorum*, *R. × pulchrum*, and *R. ovatum* exhibited rapid declines in F_v_/F_m_ under heat stress, followed by substantial recovery after a 7 d rehabilitation period. In contrast, the PSII efficiency of *R. latoucheae* remained consistently low both before and after stress, with no significant change. The PSII responses of *R*. × pulchrum and *R*. *liliiflorum* were well aligned with their F_v_/F_m_ dynamics, demonstrating significant recovery upon return to normal conditions, which indicates pronounced sensitivity of their PSII reaction centers to high temperatures. This observation is consistent with the findings of Percival et al. (2005), who reported greater heat stress tolerance in leaves of evergreen taxa compared to deciduous trees [26], further supporting the established view that PSII activity serves as a critical indicator of plant thermotolerance [27]. Notably, NPQ, qP, and Y(NPQ) recovered to or exceeded pre-stress levels after 7 d in most taxa, with this effect being particularly pronounced in *R. ovatum*, *R. latoucheae*, and *R. × pulchrum*. This suggests a heat-induced upregulation of photoprotective mechanisms [28,29]. In contrast, interspecific differences in qN and Y(NO) after recovery were minimal. Overall, these results indicate that *R. × pulchrum* and *R. ovatum* possess superior capacity for photosynthetic functional recovery following heat stress, with *R. × pulchrum* showing the most rapid response. *R. latoucheae* maintained consistently high and stable values across all measured photosynthetic parameters, suggesting constitutive thermotolerance. These interspecific differences likely reflect divergent evolutionary adaptations to heat stress and variation in the efficiency of photosystem repair mechanisms [30,31].

As the primary gateway for gas exchange in plants, dynamic regulation of stomatal morphology and function represents a key adaptive strategy to heat stress [32]. All eight *Rhododendron* taxa examined in this study exhibited typical responses, including stomatal closure, reduced stomatal aperture, and increased stomatal density under high-temperature conditions. These observations align with findings reported by Cheng et al. [21] in maize, suggesting a conserved mechanism across species to minimize transpirational water loss and lower leaf temperature. Significant reductions in stomatal length were observed in *R. × pulchrum*, *R. kiangsiense*, *R. latoucheae*, and *R. ovatum* following heat stress. Among these, *R. × pulchrum* and *R. latoucheae* displayed remarkable recovery capacity, whereas *R. simiarum* maintained stable stomatal dimensions throughout the experiment-a trait potentially associated with its characteristic leathery leaf morphology [33]. Although stomatal closure occurred after 24 h of heat stress in all taxa, *R. fortunei* and *R. × pulchrum* had the highest stomatal sensitivity, which was the same as that of cultivar ‘Zhuangyuanhong’ (*Rh.* ‘Zhuangyuan Hong’) [34]. The rapid recovery of stomatal width in *R. × pulchrum* during the rehabilitation phase may be attributed to modulations in cell wall elasticity [35,36]. In contrast, *R. ovatum* was the only species that maintained partially closed stomata even after the 7-day recovery period. This sustained closure may be attributed to its persistently high photochemical quenching (qP) value post-recovery, which could maintain the activity of the photosynthetic electron transport chain and thereby facilitate the repair efficiency of the photosynthetic apparatus [37]. This study revealed that the stomatal density of most *Rhododendron* taxa increased under acute heat stress and decreased upon temperature recovery. This trend was consistent with the previously reported cultivar *R.* ‘Liu Qiu Hong’ but opposite to that of *R.* ‘Lan Yin’ [38]. Such varietal differences highlight species-specific strategies in response to stress. Furthermore, leaf anatomical structures in several tested *Rhododendrons* exhibited significant and reversible changes within 24 h of stress. These findings suggest that the observed responses stem from elastic adjustments in existing cells and tissues, rather than the formation of new structures. This rapid and reversible stomatal behavior may represent a key adaptive mechanism enabling *Rhododendrons* to withstand short-term heat fluctuations.

The stability of leaf anatomical structure underpins plant thermotolerance. Under elevated temperatures, all investigated taxa exhibited a consistent response characterized by thickening of palisade tissue and thinning of spongy mesophyll. This structural reorganization likely represents an adaptive mechanism to enhance photosynthetic efficiency by improving light capture and reducing transpirational water loss through decreased intercellular airspace [9,39]. Notably, *R. latoucheae* and *R. kiangsiense* maintained a stable palisade to spongy tissue ratio under stress, aligning with findings reported by Li et al. [34], indicating higher anatomical resilience in these two germplasms. In contrast, *R. × pulchrum* exhibited pronounced structural plasticity during stress yet demonstrated exceptional regenerative capacity during recovery, suggesting an efficient tissue reorganization mechanism [38]. Significant reductions in epidermal thickness were observed in *R. ovatum* and *R. fortunei*, consistent with reports in the cultivar *Rhododendron* ‘Fen Zhenzhu’, which may facilitate heat dissipation at the cost of reduced protective capacity [34]. Conversely, *R. liliiflorum* achieved complete restoration of epidermal integrity after recovery, highlighting its superior capacity for structural repair [40]. However, the changes in tissue structure and cells observed in this study did not originate from the generation of new cells, but rather reflected a rapid morphological adaptation and remodeling of the existing tissue cells under acute heat stress.

Dynamic changes in physiological indicators reflect the capacity for osmotic adjustment and antioxidant defense under high-temperature stress. *R. oldhamii* exhibited the most pronounced increases in EL and MDA content, indicating severe membrane damage and heightened sensitivity to heat stress, which is consistent with its observed Grade III phenotypic injury. In contrast, *R. simiarum* and *R. × pulchrum* displayed the most substantial accumulation of proline, suggesting active osmotic adjustment that contributes to cellular homeostasis under stress conditions [41,42]. Notably, *R. latoucheae* showed a sharp decline in proline content during recovery, returning to basal levels, which indicates an efficient reestablishment of metabolic equilibrium after stress removal. This rapid metabolic recovery may underlie its superior thermotolerance [32,43].

Principal component analysis (PCA) ranked the eight *Rhododendron* taxa in the following order of heat tolerance: *R. latoucheae* > *R. simiarum* > *R. oldhamii* > *R. ovatum* > *R. fortunei* > *R. liliiflorum* > *R. kiangsiense* > *R. × pulchrum*. This order reflects the integrated contribution of multiple physiological and structural indicators to overall thermotolerance. An interesting inter-subgenus divergence was observed, with taxa of the subgenus *Azaleastrum* (*R. latoucheae* and *R. ovatum*) demonstrating superior short-term heat tolerance over those of the subgenus *Tsutsusi* (*R. oldhamii* and *R. × pulchrum*), potentially due to disparities in key leaf traits like thickness and leatheriness, combined with the brief recovery period. Other studies also confirm this assumption, indicating that the leaves of the *R. × pulchrum* are relatively thin and lack leathery characteristics. They are extremely prone to water loss and tissue damage under high-temperature stress. However, their strong self-repair and regeneration capabilities are also one of the reasons for their widespread use in horticultural landscapes [44]. This study provides a preliminary understanding of the patterns of intersubgeneric variation in heat tolerance within *Rhododendron*. However, it should be noted that the 24 h heat stress treatment employed in this study has certain limitations, and the inclusion of only two taxa per subgenus indeed constrains statistical robustness at the subgenus level. Future research should focus on analyzing the response patterns of multiple taxa within each subgenus to prolonged heat stress and systematically investigate the genetic differentiation characteristics of germplasm resources from different altitudes, thereby enabling deeper insights into the underlying physiological and molecular mechanisms.

## 4. Materials and Methods

### 4.1. Plant Materials and Methods

This research used 8 taxa of *Rhododendrons* as materials, including *R. oldhamii* and *R. × pulchrum* from *R.* subg. *Tsutsusi*, *R. kiangsiense* and *R. liliiflorum* from *R.* subg. *Rhododendron*, *R. fortunei* and *R. simiarum* from *R.* subg. *Hymenanthes*, *R. latoucheae* and *R. ovatum* from *R.* subg. *Azaleastrum*. Specifically, *R. oldhamii* is a semi-evergreen shrub inhabiting montane thickets at approximately 2800 m elevation, with thinly leathery leaves and slightly revolute margins; *R. × pulchrum*, a natural hybrid between *R. mucronatum* and *R. scabrum*, is a semi-evergreen shrub distributed in the middle and lower reaches of the Yangtze River, characterized by non-coriaceous, pubescent leaves; *R. kiangsiense* occurs on mountain slopes at around 1100 m elevation as a shrub with coriaceous leaves and slightly revolute margins; *R. liliiflorum* is an evergreen shrub found in open montane forests or thickets, bearing coriaceous leaves; *R. fortunei*, an evergreen shrub or small tree, grows on sunny ridges or in forest understories at 620–2000 m elevation, with thickly coriaceous, slightly pubescent, and moderately glossy leaves; *R. simiarum* is an evergreen shrub inhabiting slopes within forests at 500–1800 m elevation, featuring thickly coriaceous leaves with a thin indumentum on the abaxial surface; *R. latoucheae*, an evergreen shrub or small tree, grows in mixed forests at 1000–2000 m elevation, with glossy, coriaceous, and glabrous leaves on both surfaces; and *R. ovatum* is an evergreen shrub occurring in montane thickets or open forests at 350–800 m elevation, with thinly coriaceous and moderately glossy leaves.

Academy of Sciences (Nanchang Research Center). The plant materials consisted of three-year-old seedling potted plants uniformly cultivated in the *Rhododendron* greenhouse. All plants were potted in containers with a top diameter of 12 cm and a height of 18 cm, each filled with 2 kg of cultivation substrate having a water-holding capacity of 65%. Three treatment groups were established, including a 25 °C control group, with 18 pots prepared for each *Rhododendron* taxa. A destructive sampling strategy was adopted: plants from each treatment group were divided into two portions—one for non-destructive chlorophyll fluorescence measurements and the other for leaf sampling to analyze stomatal characteristics, anatomical structure, and physiological indicators. Each measurement for every treatment was conducted with three biological replicates. Based on preliminary experiments and with reference to [44], 40 °C was determined to be a suitable stress temperature. In the formal experiment, after implementing a three-day uniform watering regimen for the plants scheduled for treatment, they were transferred to a high-temperature growth chamber set at 40 °C (with a photosynthetic photon flux of 150 μmol·m^−2^·s^−1^, a photoperiod of 14 h light/10 h dark, and 85% relative humidity) for a 24 h stress treatment. Subsequently, the plants were moved to a 25 °C cultivation chamber for a 7-day recovery period before final sample collection.

### 4.2. Phenotypic Observation

Leaf damage severity was quantified using ImageJ software (version 1.54p, National Institutes of Health, Bethesda, MD, USA), following the methodology established by Li et al. [34]. We classified the severity of heat injury to *Rhododendron* leaves into 4 levels. Grade I was characterized by curled young leaves, with fewer than 10% showing symptoms such as wilting, drooping, or shriveling. Grade II involved 10–30% of leaves exhibiting these symptoms, plus browning at the leaf margins. Grade III corresponded to 30–60% of leaves having symptoms such as wilting, drooping, shriveling, and withering. Grade IV was defined as more than 60% of the leaves being severely affected by wilting, shriveling, and extensive necrosis.

### 4.3. Chlorophyll Fluorescence Imaging

For chlorophyll fluorescence detection, leaf samples were dark-adapted for 15 min prior to imaging using an IMAGING-PAM chlorophyll fluorometer (Walz, Effeltrich, Germany). The maximum quantum efficiency of photosystem II (F_v_/F_m_), photochemical quenching coefficient (qP), non-photochemical quenching coefficient (qN), actual photochemical efficiency of PSII [Y(II)], apparent photosynthetic electron transport rate (ETR), quantum yield of non-regulated energy dissipation [Y(NO)], quantum yield of regulated energy dissipation [Y(NPQ)], and non-photochemical quenching (NPQ) were determined using the Imaging WinGegE software (version 1.54p, National Institutes of Health, Bethesda, MD, USA) [45].

### 4.4. Morphological and Microscopic Observation

To evaluate the effects of high-temperature stress on *Rhododendron* leaves, comparative observations of stomatal morphology and leaf microstructure were conducted between control and stress-treated plants. Stomatal parameters were quantified using an optical microscope (Olympus, Tokyo, Japan) by examining the abaxial epidermis. Leaf tissue sections were prepared following established protocols. Samples were fixed, dehydrated, and embedded in paraffin for longitudinal sectioning. The sections were then stained with safranin-fast green and imaged under the same optical microscope. Tissue layer thickness was measured using ImageJ software [46].

### 4.5. Determination of Physiological Parameter

The leaf relative water content (RWC) was determined using the soaking–drying method. For electrolyte leakage (EL), the protocol described by Wei et al. [47] was followed, though with minor modifications. Briefly, fresh leaf samples were immersed in 40 mL of deionized water and agitated at 200 rpm for two hours. Then, the initial conductivity of the bathing solution (C_1_) and the control deionized water (CK_1_) were measured. The samples were autoclaved at 100 °C for 10 min, cooled to room temperature, and the final conductivity of the solution (C_2_) and control (CK_2_) was recorded. EL was calculated as follows: EL (%) = [(C_1_ − CK_1_)/(C_2_ − CK_2_)] × 100%. The concentrations of malondialdehyde (MDA) and proline (Pro) were quantified using commercial assay kits from the Nanjing Jiancheng Bioengineering Institute (Nanjing, China) following the manufacturer’s protocols. Specifically, MDA content was determined by measuring absorbance at 532 nm and 600 nm, with the linear regression equation calculated as A = aC + b. Proline content was measured at 520 nm absorbance, using L-proline standard solutions of known concentrations to establish a standard curve with the linear regression equation A = aC + b.

### 4.6. Statistical Analysis

All data were analyzed using SPSS version 25 software for one-way analysis of variance (ANOVA) and Duncan’s multiple range test. *p* < 0.05 indicated significant differences. Principal component analysis (PCA) was performed using the PCA module in SPSS version 25 to standardize and reduce the dimensionality of the measured indicators, followed by the application of a membership function method to comprehensively evaluate the heat tolerance of different *Rhododendron* taxa [48]. The specific procedure was as follows:

(1) Membership value of each standardized indicator:

U(Xj) = (Xj − Xmin)/(Xmax − Xmin)

(2) Weight of each composite indicator:

$$\omega i=P_{j}/\sum_{j-1}^{n}P_{j}$$

(3) Comprehensive heat tolerance D value:

$$D=\sum_{j-1}^{n}\left(U(X)\times \omega_{i}\right)$$

## 5. Conclusions

This study demonstrates significant interspecific variation in heat tolerance among eight *Rhododendron* taxa, primarily determined by their photosynthetic stability, structural integrity, and physiological resilience. *R. latoucheae* exhibited the strongest constitutive thermotolerance, maintaining stable photosynthetic performance (F_v_/F_m_), minimal membrane damage (low EL and MDA), and rapid metabolic recovery (proline reduction). In contrast, *R. × pulchrum* and *R. oldhamii* were highly sensitive but differed in recovery capacity; the former showed strong regenerative ability, while the latter sustained irreversible damage. Stomatal regulation and leaf anatomical adjustments (e.g., palisade thickening, epidermal thinning) were ubiquitous but species-specific in magnitude and reversibility. Subgenus level analysis revealed that under controlled experimental conditions, the Azaleastrum subgenus exhibited superior short-term heat stress tolerance, which may be attributed to advantageous leaf structural traits and physiological adaptation mechanisms. This study provides valuable physiological and structural insights for the selection of *Rhododendron* germplasm with short-term heat tolerance and establishes a preliminary theoretical foundation for breeding heat-resistant cultivars. It should be noted, however, that the study’s conclusions are subject to certain limitations, as each subgenus was represented by only two taxa and the experiment employed a short-term heat stress model. Further studies should involve more taxa across subgenera and implement prolonged stress treatments to enable more systematic analysis of the heat tolerance mechanisms in *Rhododendron*.

## Figures and Tables

**Table 1:** Morphological performance of eight *Rhododendron* taxa under control, heat stress, and 7 d after recovery.

Variety	CK	Heat Stress	Recovery 2 d	Recovery 4 d	Recovery 7 d
*R. oldhamii*	I	III	IV	IV	III
*R. × pulchrum*	I	III	IV	III	III
*R. kiangsiense*	I	II	III	II	I
*R. liliiflorum*	I	II	III	II	I
*R. fortunei*	I	II	II	I	I
*R. simiarum*	I	II	II	I	I
*R. latoucheae*	I	II	I	I	I
*R. ovatum*	I	II	I	I	I

**Table 2:** Ultrastructure of leaf epidermal cells of eight *Rhododendron* taxa under control, heat stress, and after 7 d recovery. Data represent mean ± SD (*n* = 3). Different lowercase letters indicate significant differences among taxa under the same treatment based on one-way ANOVA followed by Duncan’s multiple range test (*p* < 0.05).

Variety	Treatment	Stomatal Length (μm)	Stomatal Width (μm)	Stomatal Area (μm^2^)	Stomatal Density (stomata/mm^2^)
*R. oldhamii*	CK	23.13 ± 0.89 a	14.43 ± 0.40 a	270.20 ± 7.20 a	300 ± 5 c
	Heat stress	19.86 ± 0.32 c	11.57 ± 1.61 b	223.85 ± 6.75 b	400 ± 6 a
	Recovery 7 d	21.48 ± 0.36 b	13.50 ± 0.27 ab	242.68 ± 29.50 ab	333 ± 5 b
*R.× pulchrum*	CK	23.87 ± 0.90 a	10.38 ± 0.71 a	182.91 ± 3.38 a	267 ± 5 c
	Heat stress	19.19 ± 0.37 c	8.83 ± 0.92 b	139.40 ± 0.53 c	400. ± 4 a
	Recovery 7 d	20.68 ± 0.79 b	9.83 ± 0.27 b	169.37 ± 0.98 b	333 ± 5 b
*R. kiangsiense*	CK	20.56 ± 0.26 a	11.43 ± 0.32 a	215.87 ± 2.66 a	300 ± 4 c
	Heat stress	16.75 ± 0.20 b	10.11 ± 0.58 b	136.04 ± 1.43 c	400 ± 2 a
	Recovery 7 d	16.80 ± 0.12 b	10.15 ± 0.07 b	145.68 ± 3.29 b	333 ± 7 b
*R. liliiflorum*	CK	18.94 ± 0.73 a	10.18 ± 0.16 a	152.44 ± 6.04 a	300 ± 2 c
	Heat stress	18.60 ± 0.10 a	8.08 ± 1.04 b	130.46 ± 8.45 b	433 ± 3 a
	Recovery 7 d	18.78 ± 0.07 a	10.39 ± 0.22 a	144.90 ± 2.13 a	333 ± 1 b
*R. fortunei*	CK	18.73 ± 1.07 a	11.86 ± 1.42 a	195.17 ± 3.48 a	300 ± 2 c
	Heat stress	18.15 ± 0.89 a	8.75 ± 1.22 b	145.62 ± 2.57 c	400 ± 3 a
	Recovery 7 d	18.12 ± 0.75 a	9.50 ± 0.60 b	159.10 ± 4.59 b	367 ± 2 b
*R. simiarum*	CK	24.55 ± 1.21 a	13.38 ± 0.47 a	245.42 ± 4.83 a	333 ± 2 b
	Heat stress	23.24 ± 0.16 a	11.73 ± 0.16 c	221.87 ± 1.63 c	367 ± 3 a
	Recovery 7 d	23.85 ± 0.01 a	12.48 ± 0.27 b	230.47 ± 0.17 b	367 ± 5 a
*R. latoucheae*	CK	29.03 ± 0.35 a	15.72 ± 0.10 a	332.41 ± 2.23 a	267 ± 6 b
	Heat stress	23.74 ± 0.25 c	13.98 ± 0.75 b	243.24 ± 1.03 c	367 ± 2 a
	Recovery 7 d	26.20 ± 0.72 b	14.31 ± 0.41 b	280.24 ± 6.57 b	364 ± 3 a
*R. ovatum*	CK	26.91 ± 1.65 a	16.62 ± 0.32 a	332.21 ± 5.53 a	300 ± 2 b
	Heat stress	22.23 ± 1.95 b	14.45 ± 0.23 b	258.27 ± 5.90 c	367 ± 5 a
	Recovery 7 d	24.96 ± 0.52 ab	14.76 ± 0.48 b	285.93 ± 10.46 b	300 ± 3 b

**Table 3:** Leaf anatomical structure of eight *Rhododendron* taxa under control, heat stress, and after 7 d recovery. Data represent mean ± SD (*n* = 3). Different lowercase letters indicate significant differences among taxa under the same treatment based on one-way ANOVA followed by Duncan’s multiple range test (*p* < 0.05).

Variety	Treatment	Upper Epidermis Thickness (μm)	Lower Epidermis Thickness (μm)	Palisade Mesophyll Thickness (μm)	Spongy Mesophyll Thickness (μm)	Ratio of Palisade Thickness to Spongy Thickness
*R. oldhamii*	CK	17.74 ± 0.35 a	18.54 ± 0.57 a	43.44 ± 0.55 c	68.96 ± 0.63 a	0.63 ± 0.01 c
	Heat stress	12.79 ± 1.00 c	13.25 ± 0.53 c	62.44 ± 0.88 a	56.90 ± 0.51 c	1.10 ± 0.02 a
	Recovery 7 days	15.02 ± 0.70 b	14.60 ± 0.14 b	53.20 ± 0.57 b	65.31 ± 1.24 b	0.81 ± 0.02 b
*R. × pulchrum*	CK	18.58 ± 0.80 a	15.82 ± 0.39 a	38.31 ± 0.36 c	77.77 ± 1.18 a	0.49 ± 0.01 c
	Heat stress	14.82 ± 0.11 b	10.16 ± 0.10 c	57.44 ± 0.42 a	56.72 ± 0.77 c	1.01 ± 0.01 a
	Recovery 7 days	17.74 ± 0.90 a	12.44 ± 0.07 b	41.12 ± 0.76 b	59.50 ± 0.62 b	0.69 ± 0.01 b
*R. kiangsiense*	CK	26.16 ± 0.59 a	12.68 ± 0.64 a	115.57 ± 1.01 c	123.32 ± 1.11 a	0.94 ± 0.01 b
	Heat stress	23.61 ± 0.51 b	9.70 ± 0.14 c	123.04 ± 1.00 a	111.32 ± 1.37 b	1.11 ± 0.02 b
	Recovery 7 days	25.34 ± 1.13 a	10.52 ± 0.27 b	117.79 ± 0.81 b	122.63 ± 1.08 a	0.96 ± 0.01 b
*R. liliiflorum*	CK	30.83 ± 0.82 a	30.73 ± 0.83 a	104.98 ± 1.57 b	163.11 ± 2.30 a	0.64 ± 0.02 c
	Heat stress	27.96 ± 0.87 b	19.13 ± 0.84 c	115.80 ± 0.96 a	122.73 ± 1.42 c	0.94 ± 0.02 a
	Recovery 7 days	29.57 ± 1.01 ab	27.71 ± 0.45 b	106.46 ± 1.06 b	146.76 ± 1.99 b	0.73 ± 0.02 b
*R. fortunei*	CK	19.87 ± 0.92 a	14.47 ± 0.25 a	92.81 ± 1.82 c	140.72 ± 0.30 a	0.66 ± 0.01 c
	Heat stress	14.43 ± 0.23 c	11.50 ± 0.64 c	103.11 ± 0.46 a	122.44 ± 0.76 c	0.84 ± 0.01 a
	Recovery 7 days	17.09 ± 1.70 b	12.80 ± 0.72 b	96.52 ± 1.32 b	132.12 ± 1.15 b	0.73 ± 0.01 b
*R. simiarum*	CK	30.01 ± 0.65 a	27.16 ± 1.27 a	56.01 ± 0.20 c	145.27 ± 1.11 a	0.39 ± 0.01 c
	Heat stress	26.50 ± 0.89 c	16.34 ± 0.94 c	73.33 ± 2.19 a	133.21 ± 3.08 c	0.55 ± 0.03 a
	Recovery 7 days	28.14 ± 0.74 b	21.01 ± 0.39 b	64.08 ± 0.27 b	140.58 ± 0.49 b	0.46 ± 0.01 b
*R. atoucheae*	CK	12.92 ± 0.60 a	17.20 ± 1.16 a	80.36 ± 0.05 c	164.96 ± 1.52 a	0.49 ± 0.01 c
	Heat stress	9.63 ± 0.06 b	14.26 ± 0.49 b	106.92 ± 1.66 a	141.35 ± 1.49 c	0.76 ± 0.00 a
	Recovery 7 days	12.85 ± 0.86 a	15.29 ± 0.29 b	96.38 ± 0.87 b	158.05 ± 0.55 b	0.64 ± 0.01 b
*R. ovatum*	CK	14.13 ± 0.64 a	13.03 ± 0.43 a	75.74 ± 2.26 c	130.65 ± 1.69 a	0.58 ± 0.02 c
	Heat stress	10.05 ± 0.51 c	9.60 ± 0.31 c	100.43 ± 1.87 a	98.25 ± 1.24 c	1.02 ± 0.03 a
	Recovery 7 days	12.01 ± 0.28 b	10.94 ± 0.34 b	85.84 ± 1.37 b	110.88 ± 1.93 b	0.77 ± 0.02 b

**Table 4:** Eigenvectors and contributions of thermotolerance indicators in eight *Rhododendron* taxa.

Index	Principal Component					
	1	2	3	4	5	6
F_v_/F_m_	0.43	0.37	−0.57	0.40	0.34	−0.16
Y(II)	0.80	−0.06	−0.32	−0.03	0.40	−0.10
NPQ	−0.18	0.64	−0.24	0.61	−0.18	−0.10
qP	0.74	0.31	−0.34	−0.07	0.23	0.17
qN	−0.47	0.71	−0.35	0.24	−0.06	−0.02
Y(NPQ)	−0.54	0.62	−0.28	0.36	−0.19	−0.14
Y(NO)	−0.20	−0.82	0.32	−0.11	0.24	0.05
ETR	0.87	0.22	−0.22	−0.18	0.07	−0.16
Stomatal length	0.14	0.73	0.47	−0.27	−0.15	0.17
Stomatal width	0.07	0.73	0.63	−0.13	0.00	0.02
Stomatal area	0.05	0.75	0.60	−0.21	0.02	0.08
Stomatal density	−0.67	−0.42	−0.12	0.38	0.03	0.38
Upper epidermis thickness	0.55	−0.52	−0.19	0.15	−0.48	0.16
Lower epidermis thickness	0.61	−0.02	−0.08	0.14	−0.50	0.47
Palisade mesophyll thickness	0.06	−0.56	0.41	0.60	0.17	−0.16
Spongy mesophyll thickness	0.56	−0.06	0.44	0.53	−0.16	0.14
RWC	0.61	0.02	0.54	0.27	−0.12	−0.32
EL	−0.65	−0.09	0.40	0.04	−0.03	−0.30
MDA	−0.27	0.34	0.15	0.24	0.63	0.53
Pro	−0.45	−0.02	−0.63	−0.42	−0.21	0.00
Eigenvalue	5.24	4.76	3.22	2.04	1.47	1.07
Contribution rate (%)	26.20	23.79	16.10	10.19	7.34	5.33
Cumulative contribution rate (%)	26.20	50.00	66.10	76.29	83.62	88.96

**Table 5:** Comprehensive index value, membership function value, D value, and comprehensive evaluation of eight *Rhododendron* taxa.

Variety	Treatment	Membership Function						D Value		Order
		U (X_1_)	U (X_2_)	U (X_3_)	U (X_4_)	U (X_5_)	U (X_6_)			
*R. oldhamii*	CK	0.85	0.73	0.43	0.52	0.33	0.44	0.60	0.555	3
	Heat stress	0.53	0.37	0.51	0.42	0.26	1.00	0.49		
	Recovery 7 days	0.78	0.52	0.62	0.54	0.23	0.60	0.58		
*R. pulchrum*	CK	1.00	0.48	0.30	0.45	0.19	0.03	0.49	0.401	8
	Heat stress	0.35	0.09	0.56	0.00	0.38	0.33	0.28		
	Recovery 7 days	0.76	0.27	0.66	0.23	0.26	0.02	0.43		
*R. kiangsiense*	CK	0.75	0.38	0.00	0.89	0.23	0.20	0.45	0.440	7
	Heat stress	0.52	0.00	0.16	0.96	0.10	0.45	0.36		
	Recovery 7 days	0.80	0.01	0.67	0.99	0.11	0.20	0.51		
*R. liliiflorum*	CK	0.71	0.24	0.34	0.89	0.93	0.19	0.55	0.498	6
	Heat stress	0.26	0.06	0.21	0.80	0.74	0.38	0.36		
	Recovery 7 days	0.74	0.07	0.76	1.00	0.73	0.18	0.59		
*R. fortunei*	CK	0.92	0.29	0.36	0.85	0.18	0.27	0.53	0.504	5
	Heat stress	0.62	0.01	0.39	0.82	0.17	0.63	0.43		
	Recovery 7 days	0.79	0.03	0.81	0.92	0.21	0.36	0.55		
*R. simiarum*	CK	0.71	0.70	0.32	0.67	1.00	0.33	0.62	0.598	2
	Heat stress	0.43	0.46	0.33	0.74	0.62	0.44	0.49		
	Recovery 7 days	0.57	0.43	1.00	0.76	0.86	0.29	0.66		
*R.atoucheae*	CK	0.89	1.00	0.36	0.84	0.35	0.50	0.71	0.680	1
	Heat stress	0.54	0.56	0.57	0.91	0.31	0.91	0.62		
	Recovery 7 days	0.71	0.62	0.73	0.95	0.38	0.81	0.71		
*R. ovatum*	CK	0.43	0.99	0.45	0.81	0.35	0.07	0.58	0.510	4
	Heat stress	0.00	0.65	0.50	0.87	0.00	0.11	0.39		
	Recovery 7 days	0.46	0.70	0.81	0.92	0.01	0.00	0.57		

## Data Availability

All data generated or analyzed during this study are included in this published article and its Supplementary Materials Files.

## Supplementary Materials

The following supporting information can be downloaded at https://www.mdpi.com/article/10.3390/plants14233664/s1, Figure S1. (A,C) Chlorophyll fluorescence images of qP and qN in eight *Rhododendron* varieties under control, heat stress, and after 7 d of recovery. (B,D) Changes in qP and qN of eight *Rhododendron* varieties during heat stress and the 7-day recovery period; Figure S2. (A,C) Chlorophyll fluorescence images of Y(NO) and Y(NPQ) in eight *Rhododendron* varieties under control, heat stress, and after 7 d of recovery. (B,D) Changes in Y(NO) and Y(NPQ) of eight *Rhododendron* varieties during heat stress and the 7-day recovery period; Figure S3. (A,C) Changes in NPQ and ETR of eight *Rhododendron* varieties during heat stress and the 7-day recovery period. (B) Chlorophyll fluorescence images of NPQ in eight *Rhododendron* varieties under control, heat stress, and after 7 d of recovery.
