# Supplementary material for: Integrating Chlorophyll Fluorescence with Anatomical and Physiological Analyses Reveals Interspecific Variation in Heat Tolerance Among Eight Rhododendron Taxa
Source: Plants (Basel). 2025 Dec 1;14(23):3664. doi: 10.3390/plants14233664 (PMC12694074; doi:10.3390/plants14233664)
Supplement: Supplementary file 1 [file plants-14-03664-s001.zip › plants-3987301-supplementary.pdf]

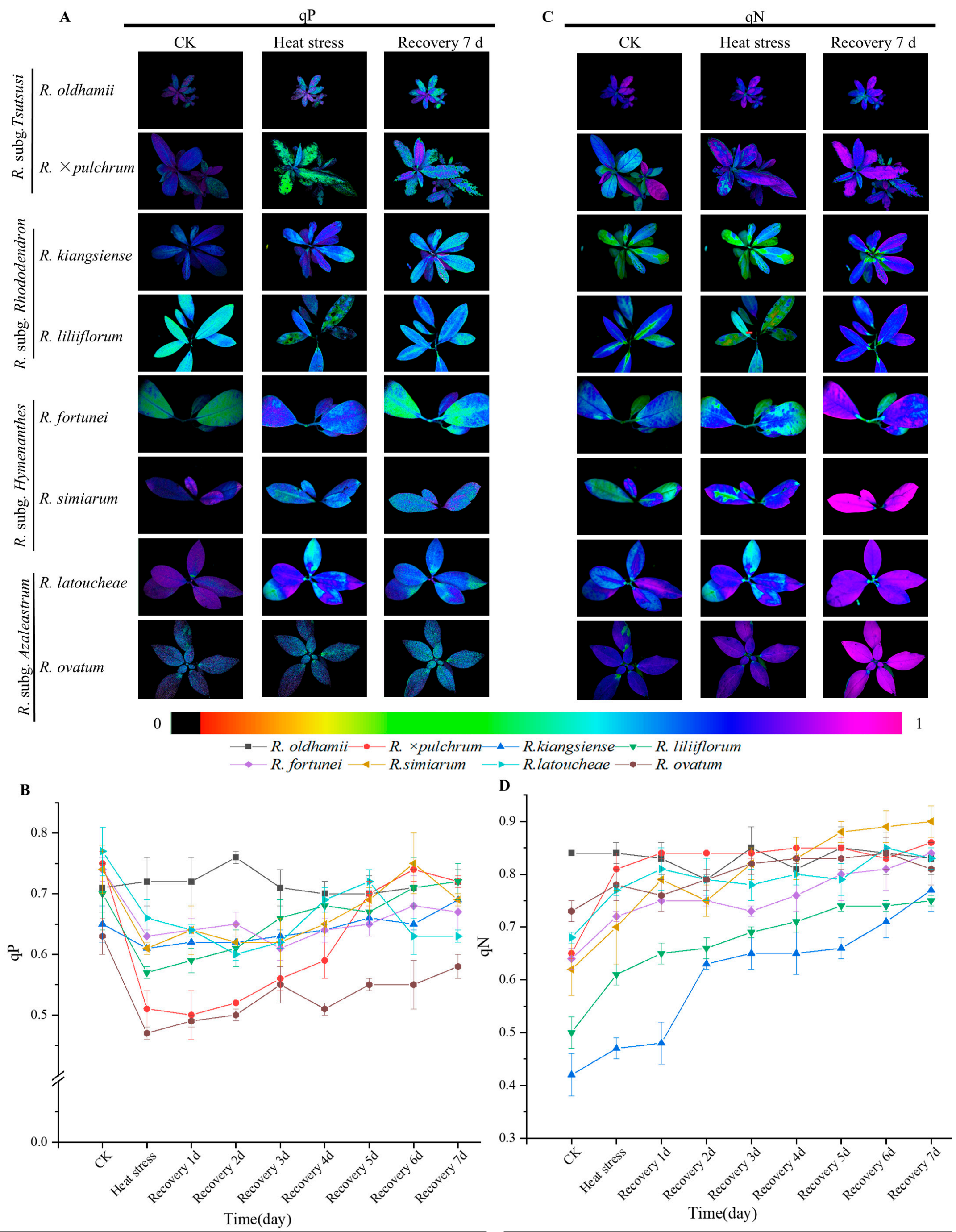

Time(day) / Variety	CK	Heat stress	Recovery 1 d	Recovery 2 d	Recovery 3 d	Recovery 4 d	Recoery 5 d	Recover y 6 d	Recovery 7 d
.oldhamii	a	ab	b	ab	ab	ab	ab	ab	ab
.×pulchrum	a	d	d	d	c	c	b	a	ab
.kiangsiense	b	d	cd	cd	cd	bc	b	b	a
.liliiflorum	a	c	c	bc	ab	ab	a	a	a
.fortunei	a	c	bc	bc	c	c	bc	b	bc
.simiarum	ab	d	d	d	d	cd	bc	c	a
.latoucheae	a	e	de	de	de	de	cd	bc	b
.ovatum	a	e	de	d	bc	cd	bc	c	b

Time(day) / Variety	CK	Heat stress	Recovery 1 d	Recovery 2 d	Recovery 3 d	Recovery 4 d	Recoery 5d	Recovery 6 d
R.oldhamii	a	ab	b	ab	ab	ab	ab	ab
R.×pulchrum	a	d	d	d	c	c	b	a
R.kiangsiense	b	d	cd	cd	cd	bc	b	b
R.liliiflorum	a	c	c	bc	ab	ab	a	a
R.fortunei	a	c	bc	bc	c	c	bc	b
R.simiarum	ab	d	d	d	d	cd	bc	c
R.latoucheae	a	e	de	de	de	de	cd	bc
R.ovatum	a	e	de	d	bc	cd	bc	c

Figure S1. (**A**,**C**) Chlorophyll fluorescence image qP and qN of eight *Rhododendron* taxa under control conditions, after 24 h of heat stress, and following 7 d of recovery. (**B**,**D**) Temporal changes in qP and qN of the eight taxa during heat stress and the 7 d recovery period. Data represent mean ±

SD (n = 3). Different letters indicate significant differences among taxa at each time point based on one-way ANOVA followed by Duncan's multiple range test (p < 0.05).

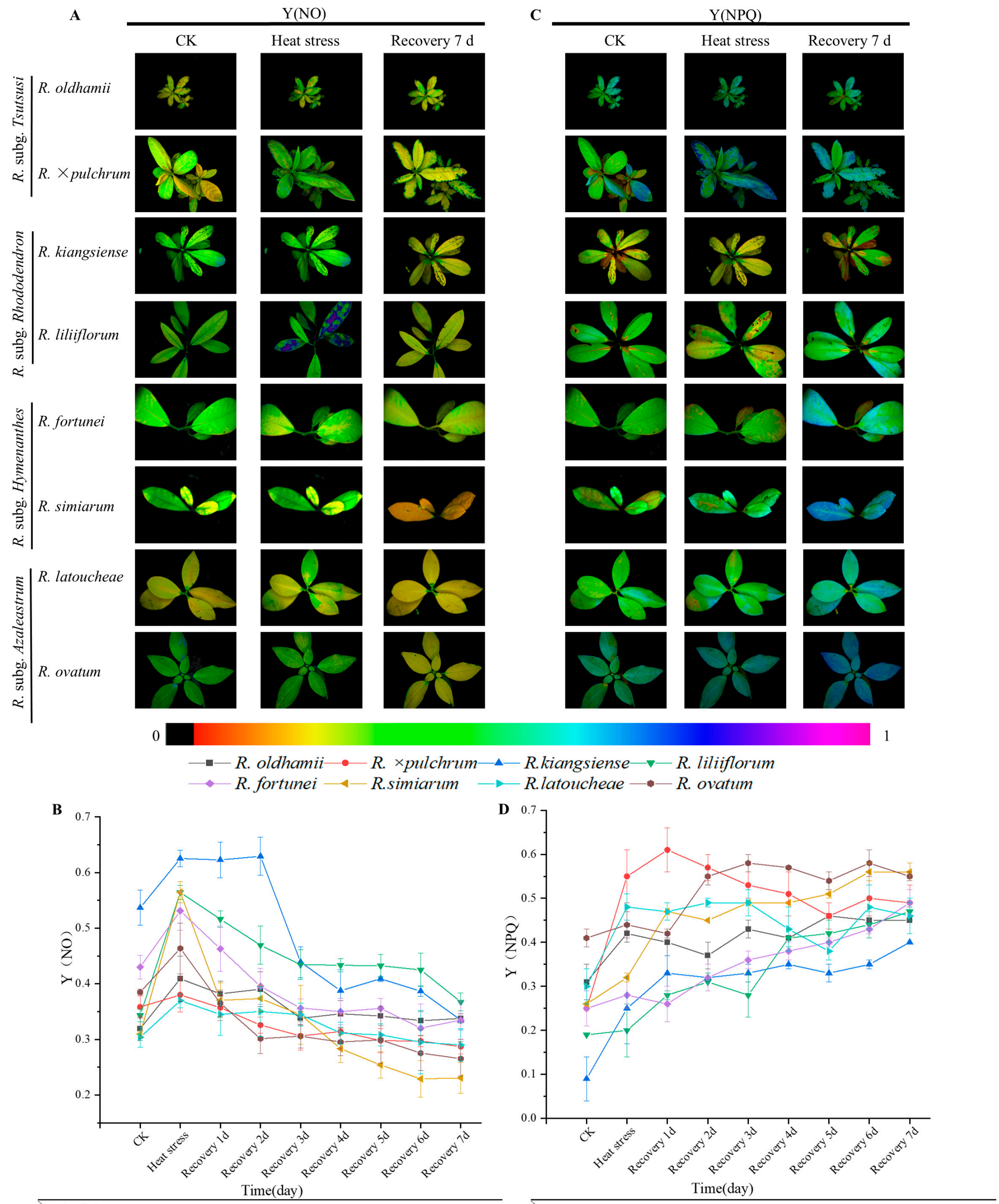

Time(day) / Variety	CK	Heat stress	Recovery 1 d	Recovery 2 d	Recovery 3 d	Recovery 4 d	Recoery 5 d	Recovery 6 d	Recovery 7 d
R.oldhamii	c	a	ab	a	c	bc	bc	c	c
R.×pulchrum	e	a	ab	bc	cde	cd	cde	cde	de
R.kiangsiense	b	a	a	a	c	d	cd	d	e
R.liliiflorum	e	a	b	c	d	d	d	d	e
R.fortunei	cd	a	b	c	cd	cd	cd	d	d
R.simiarum	cd	a	b	b	bc	de	ef	f	f
R.latoucheae	b	a	a	a	c	d	cd	d	e
R.ovatum	b	a	b	c	c	c	c	c	c

Time(day) / Variety	CK	Heat stress	Recovery 1 d	Recovery 2 d	Recovery 3 d	Recovery 4 d	Recoery 5 d	Recovery 6 d	Recovery 7 d
R.oldhamii	d	ab	bc	c	ab	abc	a	ab	a
R.×pulchrum	e	abc	a	bc	bc	bcd	d	cd	cd
R.kiangsiense	d	c	b	b	b	ab	b	ab	a
R.liliiflorum	d	d	c	c	c	b	b	ab	a
R.fortunei	f	ef	f	e	d	cd	bc	b	a
R.simiarum	f	e	cd	d	bc	bc	b	a	a
R.latoucheae	d	ab	ab	a	a	bc	b	ab	ab
R.lovatum	d	d	d	bc	ab	abc	c	a	abc

Figure S2. (**A**,**C**) Chlorophyll fluorescence image qP and qN of eight *Rhododendron* taxa under control conditions, after 24 h of heat stress, and following 7 d of recovery. (**B**,**D**) Temporal changes in qP and qN of the eight taxa during heat stress and the 7 d recovery period. Data represent mean ± SD (n = 3). Different letters indicate significant differences among taxa at each time point based on one-way ANOVA followed by Duncan's multiple range test (p < 0.05).

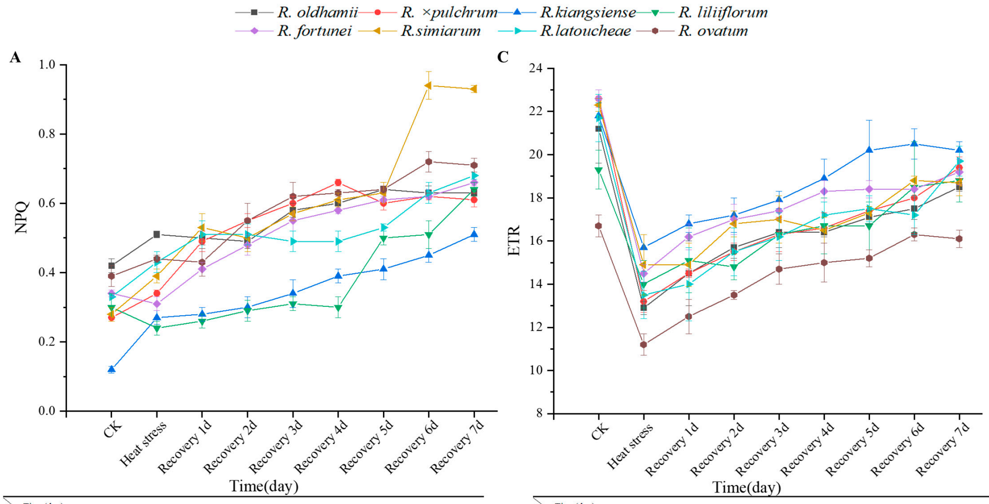

Time(day) / Variety	CK	Heat stress	Recovery 1 d	Recovery 2 d	Recovery 3 d	Recovery 4 d	Recoery 5 d	Recovery 6 d	Recovery 7 d
R.oldhamii	e	c	cd	cd	b	b	a	a	a
R.×pulchrum	e	f	d	c	b	a	b	b	b
R.kiangsiense	f	e	e	e	d	c	c	b	a
R.liliiflorum	c	e	de	cd	c	c	b	b	a
R.fortunei	c	e	de	cd	c	c	b	b	a
R.simiarum	f	e	cd	c	d	b	b	a	a
R.latoucheae	f	e	cd	cd	cd	d	c	b	a
R.ovatum	e	d	cd	c	b	b	b	a	a

Time(day) / Variety	CK	Heat stress	Recovery 1 d	Recovery 2 d	Recovery 3 d	Recovery 4 d	Recoery 5d	Recovery 6 d	Recovery 7 d
R.oldhamii	a	f	e	de	cd	cd	c	bc	b
R.×pulchrum	a	h	f	e	d	d	c	c	b
R.kiangsiense	a	e	de	d	c	c	b	b	b
R.liliiflorum	a	d	cd	cd	c	bc	bc	ab	a
R.fortunei	a	f	e	d	d	c	c	c	b
R.simiarum	a	d	d	c	c	c	c	b	b
R.latoucheae	a	f	ef	de	cd	cd	c	c	b
R.ovatum	a	f	e	d	c	c	bc	a	ab

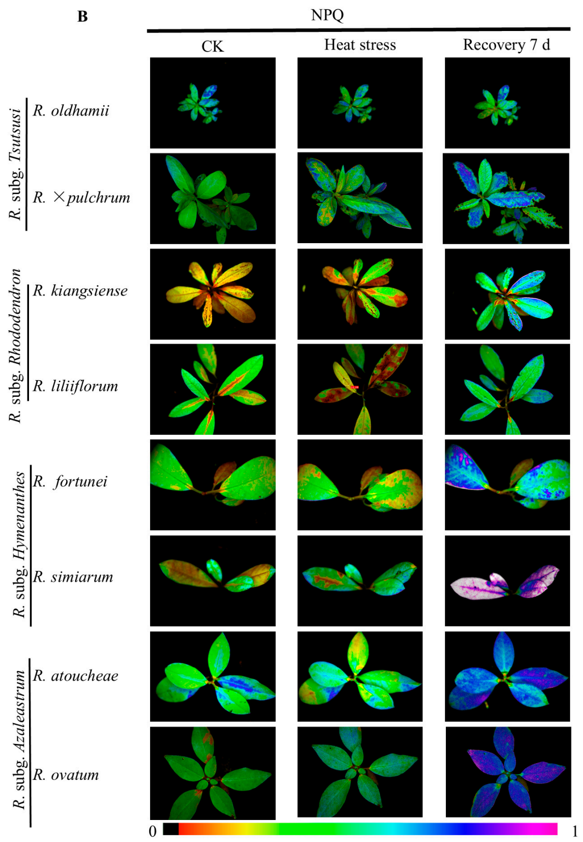

Figure S3. (**A**,**C**) Chlorophyll fluorescence image ~~NPQ~~, NPQ, and ETR of eight *Rhododendron* taxa under control conditions, after 24 h of heat stress, and following 7 d of recovery. (**B**) Temporal changes in NPQ of the eight taxa during heat stress and the 7 d recovery period. Data represent mean ± SD (n = 3). Different letters indicate significant differences among taxa at each ~~at~~ time point based on one-way ANOVA followed by Duncan's multiple range test ($p < 0.05$).
